# The negative effect of ceria on the propene selectivity for isopropanol decomposition over phosphated and phosphate-free ceria/alumina catalysts

**DOI:** 10.1186/2193-1801-2-619

**Published:** 2013-11-20

**Authors:** Hussein A Khalaf

**Affiliations:** Chemistry Department, Faculty of Science, Omar–El-Mukhtar University, P.O. 919 El-Beida, Libya

**Keywords:** Texture, Mesoporous, Acidity, Activity, Decomposition

## Abstract

Consequences of loading level of ceria (5, 10 and 20-wt%) on the structural, textural and surface acid–base properties as well as the impacts on catalytic activity towards isopropanol decomposition on pure and phosphated alumina is described. The obtained results indicated that composites mainly kept γ–alumina structure and the addition of ceria and/or phosphate has no effect on the bulk structure of alumina. Textural characteristics show that the phosphated composites have higher surface area than phosphate free samples. Pyridine adsorption as followed by FTIR indicated the presence of Lewis acid sites with different strengths. Moreover, results of gas phase decomposition of isopropanol show a systematic inhibition in propene selectivity based on ceria ratio.

**Electronic supplementary material:**

The online version of this article (doi:10.1186/2193-1801-2-619) contains supplementary material, which is available to authorized users.

## Introduction

Active alumina, as one of the most common catalyst support, has a good performance in catalysis applications due to its physical and chemical properties (Levy et al. 1968). Alumina has various properties such as high specific surface area, surface acidity and defects in their crystalline structure that are the key factor in the wide range of applications. One of the most important factors in alumina utilization is thermal stability, which is a very important issue for processes running at high temperature (Chen et al. 2001). Therefore, the addition of some additives can improve the thermal stability of alumina catalysts, hold the surface area and improve the surface acidity (Shinjoh 2006; Ozawa 2006; Ozawa & Nishio 2004; Ersoy & Gunay 2004). Phosphates have been claimed to act as a support stabilizer (Gishti et al. 1984; Abbattista et al. 1990; Khalaf et al. 2007). Gishti et al. (1984; evidenced a stable effect of phosphate by studying the phase transitions of alumina in the range 673–1323 K. However, the surface area decreased with the increasing content of phosphate in alumina, even less than the blank sample after calcinated at 1073 K. Khalaf et al. 2007 also proved no surface area stabilization effect from phosphates in the case of the transition phase (spinel) aluminas.

Ceria possesses versatile acid–base properties, depending on the nature and temperature of the pretreatment. It may have high number of basic sites of weak or medium strength (Binet et al. 1999). Martin and Duprez found the following scale for the density of basic sites of some metal oxides CeO_2_ > MgO > ZrO_2_ > Al_2_O_3_ > SiO_2_ (Martin & Duprez 1997). Binet et al. (1999) also observed that ceria can chemisorb CO or pyridine, but the band positions strongly suggest that the Lewis acidity of ceria is significantly lower than that of zirconia or titania. In contrast to Lewis basicity, the Lewis acidity would decrease upon reduction of ceria.

Ceria and ceria-based materials has been widely employed as automotive three-way catalysts (TWC) for reducing the exhaust pollutants, fuel cell processes, oxygen permeation membrane systems, exhaust combustion catalysts, and catalytic wet oxidation (Yao & Yao 1984; Dos Santos et al. 2008; Bera et al. 2003; Khalil 2007). Cerium oxide can maintain large amounts of oxygen through the easy transition between Ce(IV) and Ce(III). This property is very useful in catalysis especially in oxidation reactions (Guimaraes et al. 2003). Nevertheless, pure ceria is poorly thermostable and undergoes rapid sintering under high temperature conditions, which leads to loss of oxygen buffer capacity and deactivation of the catalysts. The combination of two metals in an oxide can lead to novel structural and electronic properties of the final oxide, consequently modifying its catalytic activity and selectivity. Ceria-alumina mixed oxides are widely used as catalytic materials (Walter & Oldfield 1989; Niu et al. 1999; Sanchez-Valente et al. 2004).

**Scheme 1:**
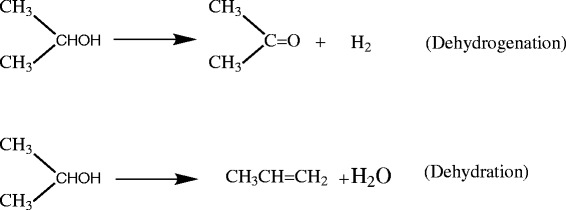
**Catalytic decomposition of isopropanol.**

Isopropanol conversion is widely investigated and lately it is used to characterize acid–base or redox properties of catalysts (Khalaf et al. 2007). The conversion of isopropanol is known to occur through two competitive pathways namely dehydrogenation and dehydration. Ai (1977) has assumed that the dehydration of isopropanol probes acid sites, whereas the dehydrogenation probes acid and base sites functioning in a concerted fashion. Isopropanol usually dehydrates to propene over acidic catalysts and dehydrogenates to acetone over basic catalysts (Scheme 1). The acid–base pairs responsible for acetone formation are strongly activated in the presence of oxygen that indicates that they are related to redox properties of the material (Abdellah et al. 1993).

In the present work, ceria-alumina as well as phosphate alumina modified with ceria have been prepared and characterized by several tools to gain an overview on the surface properties of these catalysts. In addition, the catalytic activities towards isopropanol decomposition for these composites have been explored.

## Results and discussion

### Thermal analysis

**Figure 1:**
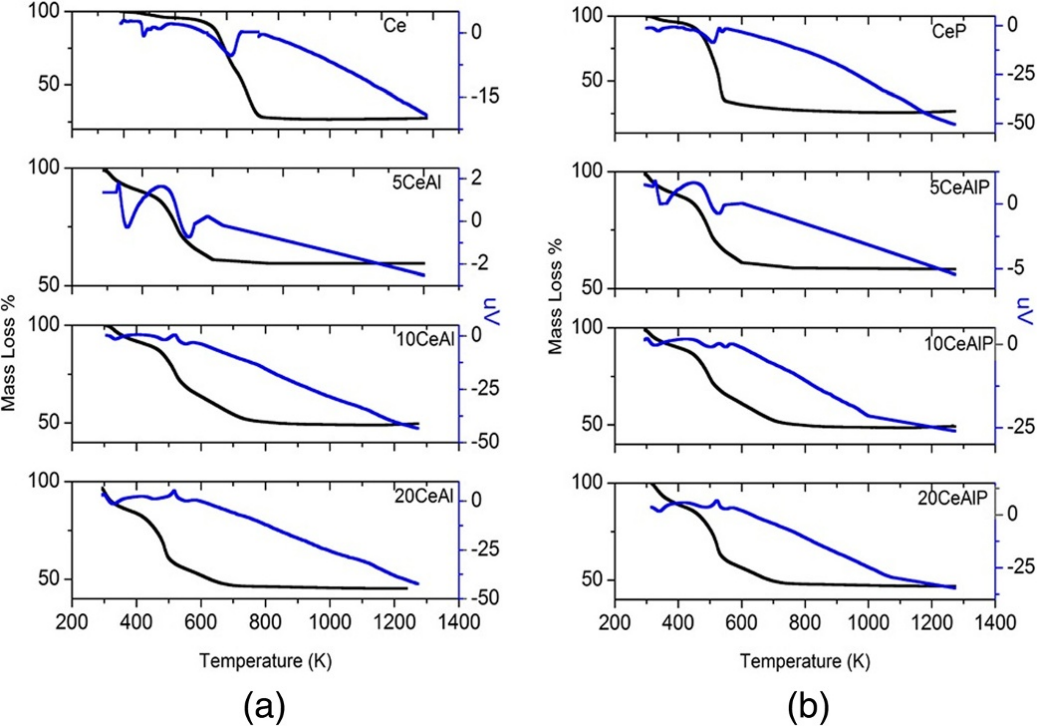
**TGA profiles for (a); non-phosphated and (b); phosphated ceria/alumina catalysts.**

The thermal behaviors of the pure alumina gel and phosphate alumina has been studied in previous paper (Khalaf et al. 2007). Figure 1 (a and b) shows the thermal events of pure ceria, phosphate and phosphate-free ceria-alumina samples. Thermal analysis results for pure ceria (Ce) exhibited the loss of physisorbed water and partial surface dehydroxylation through two steps. The first step, 4.3% mass loss, appeared at 370 K due to the loss of physisorbed water, and the other mass loss step (ca 72.4%) is the main decomposition process that occurred in a narrow temperature region (440 – 570 K). The addition of phosphate by 6 wt% into ceria gel (CeP) does not have a significant change in the amount of mass loss. DTA curve for, Ce and CeP, shows an endothermic mass loss at 340 K, corresponding to the elimination of water absorbed by crystalline ceria. Another endothermic peak around 480 K, correlated to a mass loss, that must be considered as due to the crystallization of the residual amorphous phase.

The thermal events of ceria-alumina, xCeAl, and phosphate ceria-alumina, xCeAlP, samples are shown in Figure 1. It is cleared that, the increasing in the ceria loading levels resulted in increasing in the mass loss % (39.3, 50.0 and 54.0% for 5CeAl, 10CeAl and 20CeAl samples, respectively). This is logically, due to ceria losses mass higher than alumina. As for phosphated samples, one can noticed that there is no change in the mass loss % by the comparison between phosphated and phosphate-free samples. Thus, the phosphate ions have slightly effect on the mass loss % of phosphate samples. Only the main difference between xCeAl and xCeAlP is in the wider extended region for phosphated samples.

DTA results for all samples show two endothermic peaks, the first one appeared at low temperature (about 330 K) due to the elemination of adsorbet water, and the other one appeared at temperature higher than 480 K due to the decomposition of samples.

### X-ray diffraction (XRD)

**Figure 2:**
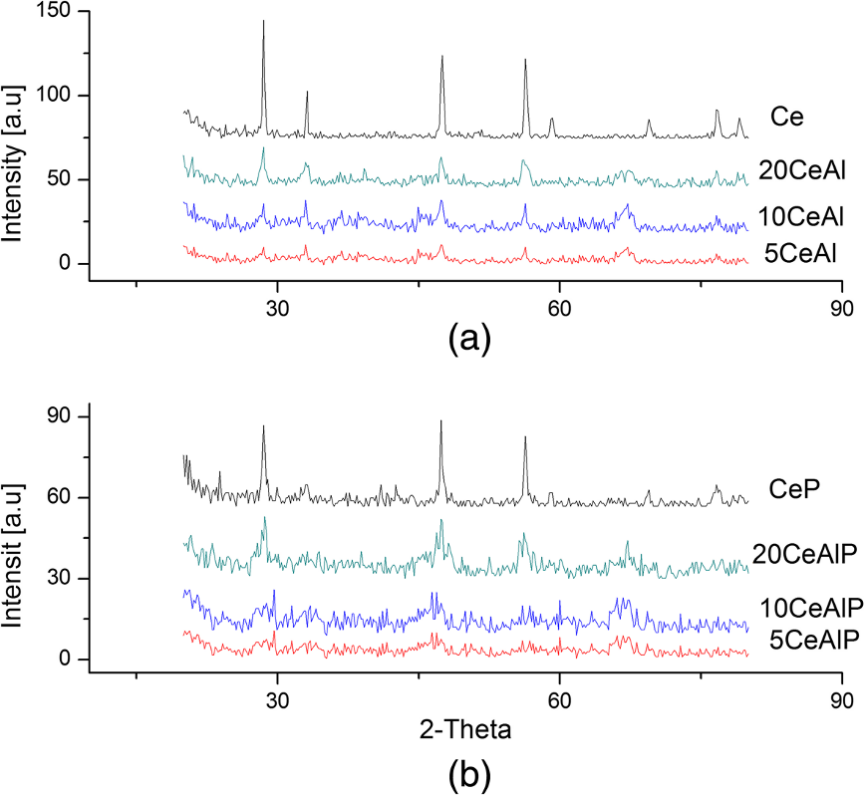
**X-ray powder diffractograms for (a); non-phosphated and (b); phosphated ceria/alumina catalysts.**

XRD patterns for tested samples were shown in Figure 2. These diffractograms indicate that the coprecipitation of ceria/alumina with different loading levels followed by calcinations causes a modification of γ-Al_2_O_3_ structure. Many researchers (Khalaf et al. 2007; López et al. 2010; Marcu et al. 2010) had investigated the crystalline structure of pure ceria and alumina. The results of alumina and phosphate alumina have been investigated in the literature (Khalaf et al. 2007). For ceria, the XRD diffractogram (Figure 2a) displayed sharp and intense peaks corresponding to cubic CeO_2_ fluorite structure as matched with the database in JCPDS (file number 04–0593) (JCPDS, International Centre for Diffraction Data, PCPDFWIN 1995). For other samples, phosphate and nonphosphated, inspecting the diffractograms and matching with the relevant ASTM standards indicate that all samples xCeAl and xCeAlP assume the γ-structure of alumina as prominent and fluorite structure for ceria as a little. These results are similar to those previously reported (Khalil 2007; Silversand et al. 1997; Johnson 1990). The cubic pattern of ceria (fluorite structure) was slightly noticed in higher loading level (20%) than other samples, but a second phase of γ-Al_2_O_3_ was also prominent at all samples. Furthermore, increasing the loading of ceria from w = 5, 10 to 20%, resulted in more increase in the degree of crystallization (higher peak intensities). The introduction of phosphate ions from ammonium salt into gel causes a slight decrease in the degree of crystallization of these samples. The crystallite size was estimated from the Scherrer equation (Klug & Alexander 1970), and the results are cited in Table 1. The smallest particle size (4.3 nm) between the present two groups of composites (xCeAl and xCeAlP) was detected for the 5CeAlP composite. For 10CeAlP and 20CeAlP samples, particle sizes were estimated as 5.8 and 7.0 nm, respectively. Particle sizes observed for the 5CeAl, 10CeAl and 20CeAl composites were 5.4, 8.1, and 12.2 nm, respectively, which is a higher than the value obtained for phosphated samples. Moreover, the obtained diffractograms for the samples at low levels revealed that fluorite-structured CeO_2_ crystallites are well dispersed on alumina surfaces. These data are agreed with that obtained from nitrogen sorption isotherms.

**Table 1:** **Nitrogen adsorption-desorption data**

Sample	S_BET_m^2^g^-1^	C_BET_	S_t_	Aver r_P_^(a)^Å	Crystallite size^(b)^(nm)
			m^2^g^-1^		
Al	187	128	193	36.3	3.3
Ce	12	6.9	11.8	15.1	44.4
5CeAl	117	98.0	114.3	20.8	5.4
10CeAl	53	74.0	51.2	19.9	8.1
20CeAl	50	58.7	48	19.3	9.2
AlP	144	129	143	42.1	4.2
CeP	12	3.5	12.1	12.1	36.2
5CeAlP	144	129	144.2	19.8	4.3
10CeAlP	105	60.4	104.2	19.5	5.8
20CeAlP	98	70.4	99	19.5	7.0

(a) Mean pore radius at the peak of the distribution curves, (b) Determined using Equation 1.

### Textural properties

**Figure 3:**
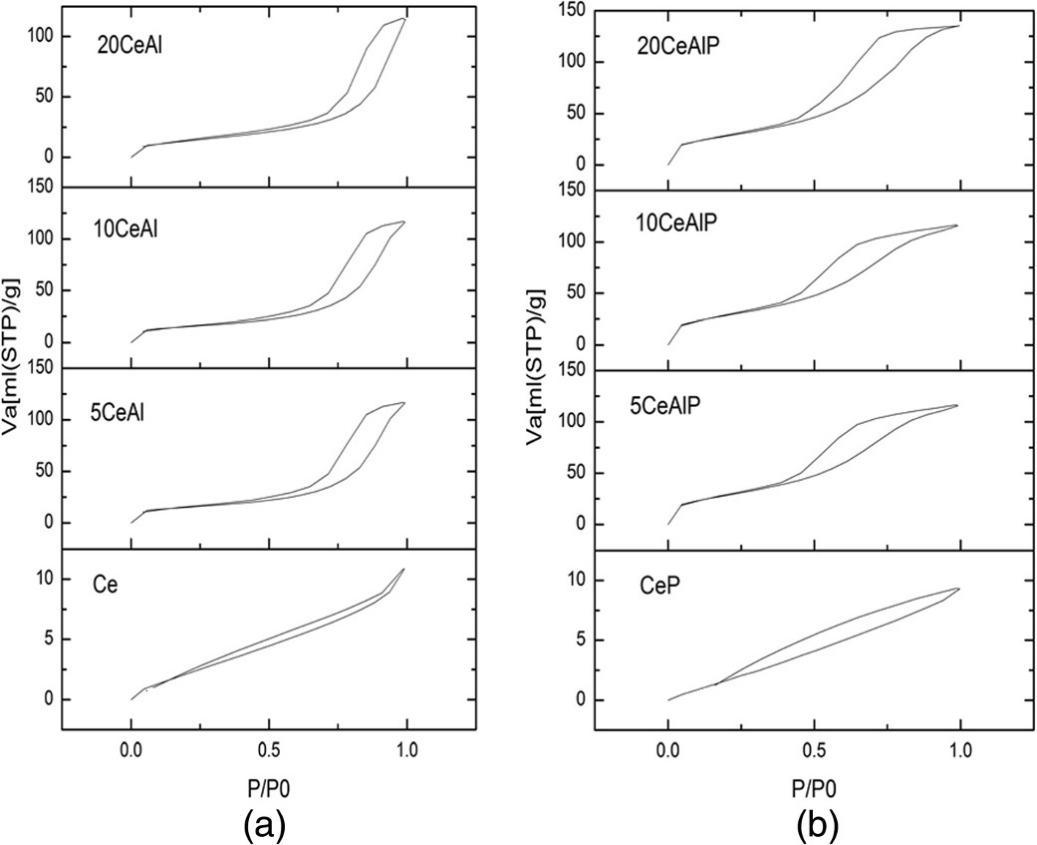
**Nitrogen sorption isotherms for (a); non-phosphated and (b); phosphated ceria/alumina catalysts,** ***V***_**a**_ **is measured at STP.**

**Figure 4:**
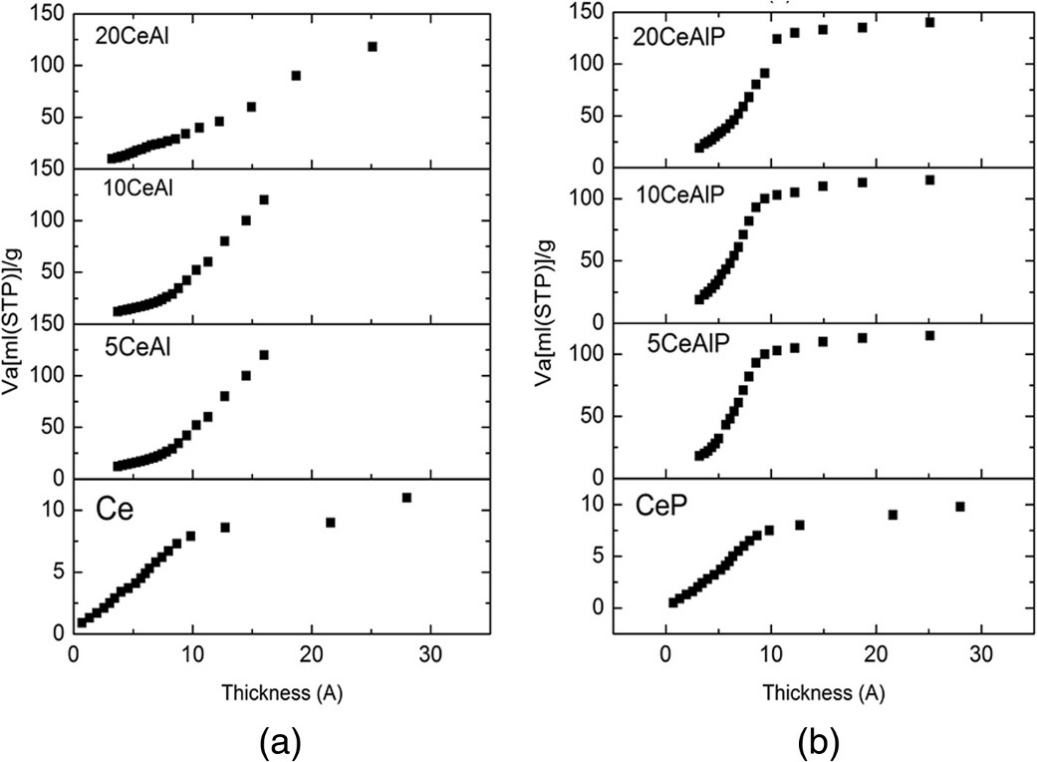
**Pore size distribution curves (a); non-phosphated and (b); phosphated ceria/alumina catalysts in which** ***r***_**p**_ **true radius and Δ** ***V***_**P**_ **· Δ** ***r***_**P**_^**-1**^ **/ cm** ^**3**^ **g** ^**-1**^ **Å** ^**-1**^ **is the ratio between the volume decrements (Δ** ***V***_**P**_ **) in cm** ^**3**^ **g** ^**-1**^ **and the decrement in pore radius (Δ** ***r***_**P**_^**-1**^ **) in Å** ^**-1**^ **.**

Nitrogen adsorption/desorption isotherms at 77 K for pure and modified samples are shown in (Figure 3a and b). From this figure, all isotherms showed Type IV profile according to BET classification (Brunauer et al. 1938), which is characteristic of porous adsorbents (Gregg & Sing 1982). The hysteresis loop can be classified as a mixed H2 and H3 types of hysteresis loops according to International Union of Pure and Applied Chemistry (IUPAC) classification, which associated with capillary condensation in mesopores materials due to the textural of inter-particle mesoporosity (Sing et al. 1985). All the samples have a close closure point at P/P_o_ = 0.4. This may actually mean that the complete monolayer formation takes place slowly and there is an effective contribution of micropores to the adsorption on the samples. This is confirmed by the pore size distribution (PSD) curves that were obtained from the desorption isotherms; see Figure 4. It also means that the capillary condensation might start from the pore size at about 3 nm.

Data obtained from isotherms and BET plots of the samples under testing are cited in Table 1. From these data, it is clear that pure alumina sample has higher specific surface area (S_BET_ = 187 m^2^g^-1^) than pure ceria (S_BET_ = 12 m^2^g^-1^) which agree with previous data (Gishti et al. 1984). The addition of ceria into alumina, xCeAl, resulted in a gradual decrease in the surface area for the samples (S_BET_ = 117, 53 and 50 m^2^g^-1^ for 5CeAl, 10CeAl and 20CeAl, respectively). For phosphated samples, there is a slightly effect of phosphate on the value of specific surface area in which the S_BET_ values for xCeAlP are higher than that of xCeAl. These data agreed with those obtained from XRD results, which complied in Table 1, in which the variation in crystallite size has an effect on the specific surface area (Khalaf 2009). Hence, the noticeable increase in the S_BET_ values for xCeAlP after phosphate addition could be attributed to the decrease in the crystallite size rather than any modification in the pore structure of the pure alumina. This result agrees with what has been reported in the literature (Larese et al. 2004).

**Figure 5:**
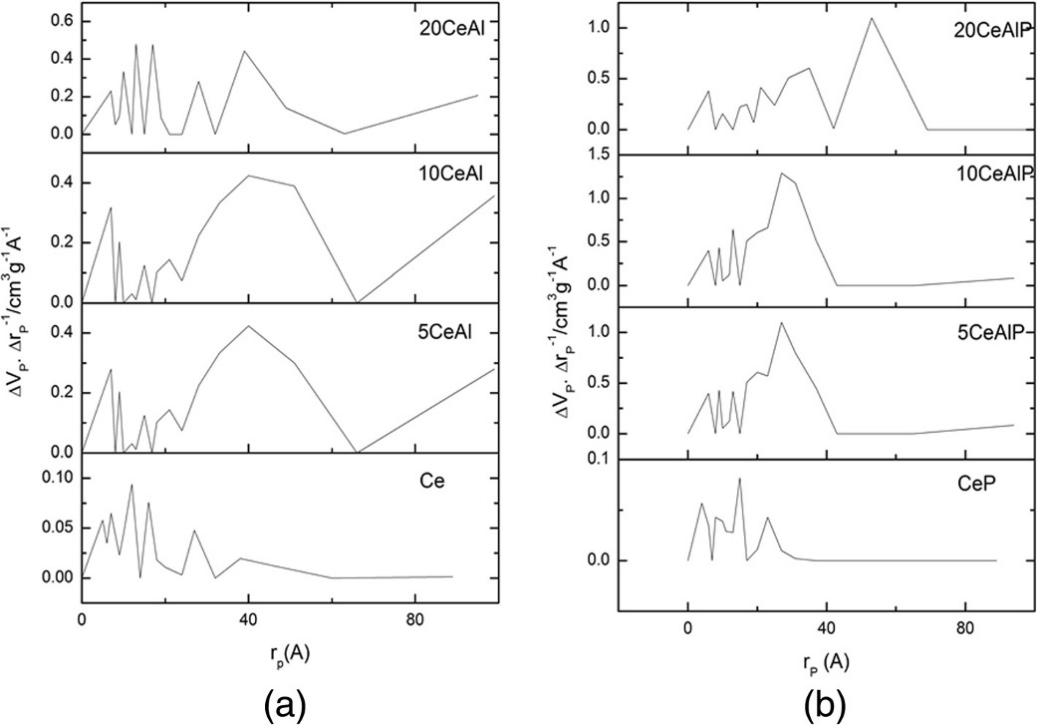
***V***_**a**_ **-** ***t*** **plots for (a); non-phosphated and (b); phosphated ceria/alumina catalysts,** ***t*** **is the thickness.**

The t-plots, constructed using the appropriate standard t-curve (Mikhail & Sheb 1970), are shown in Figure 5. From the resulting curves, one can deduce that all samples show a positive (upword) deviation in the region corresponding to capillary condensation and hence indicating the presence of mesoporosity (Mikhail & Sheb 1970). The existence of micropores was indicated by the marginal downward deviation of the corresponding t-plots in the multilayer region (Ismail & Hussein 1996). This can actually find some support from the pore size distribution curves (Figure 4). The good agreement between the S_BET_ and S_t_ values (Table 1) for all samples, reflects the higher accuracy of the BET-C determination and, consequently, the appropriateness of the reference Va-t curves (Gregg & Sing 1982).

PSD curves of the samples under study are illustrated in Figure 4. Examining these curves reveals that the addition of ceria and/or phosphate on to alumina causes a development in the porosity. In which, all samples are in micro-meso range. Moreover, the amount of mesopores in the phosphate-free samples is higher than that of phosphate samples.

### FTIR spectra of adsorbed pyridine

**Figure 6:**
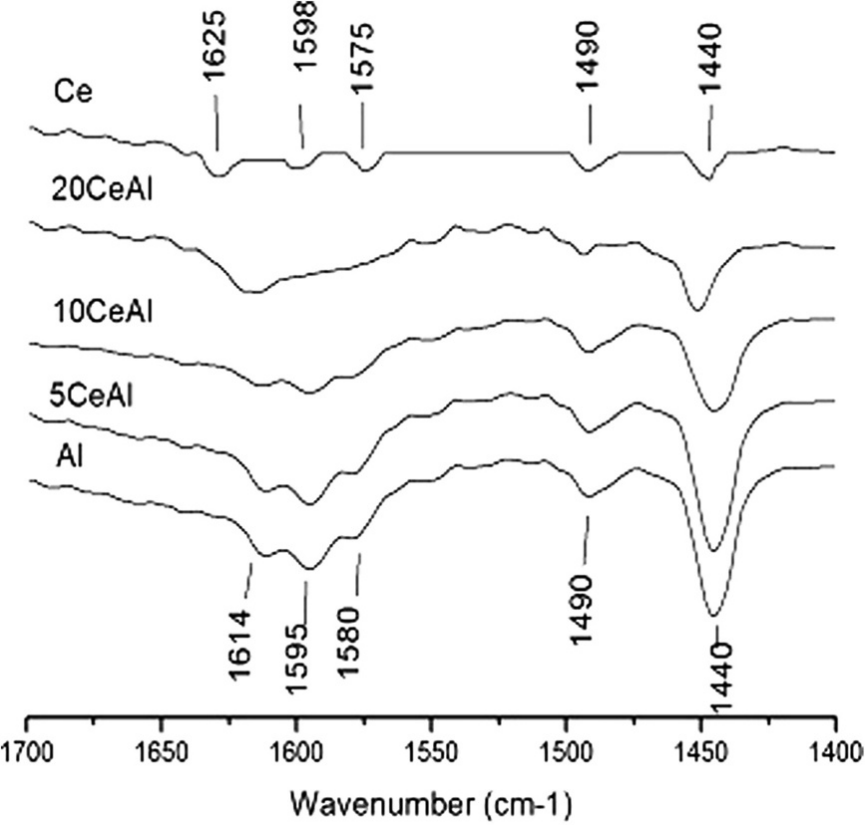
**Py adsorption at 300 K over pure and mixed oxides.**

FTIR spectrum of pyrodine (*Py*) adsorption on pure alumina (Al) is shown (Figure 6) to display five bands at 1614, 1595, 1580, 1490 and 1440 cm^-1^. These bands are assigned to hydrogen bonded and coordinately bonded *Py*. The band at ~1614 cm^-1^ is due to *Py* coordinately bonded to *Lewis* acid sites of moderate strength assigned them to tetrahedral aluminum vacancies (Khalaf et al. 2007; Mekhemer et al. 2000). While the band at 1595 cm^-1^ is due to *Py* coordinately bonded to octahedral aluminum Lewis acid sites (Khalaf et al. 2007; Mekhemer et al. 2000). The other bands: 1580, 1490 and 1440 cm^-1^ is due to *Py* species coordinated to Lewis acid sites. No observable bands around 1540–1550 cm^-1^ indicating the absence of Brönsted acid sites on alumina surface. On pure ceria (Ce), Py adsorption has given rise to five bands at 1625, 1598, 1575, 1490 and 1440 cm^-1^. Accordingly, these bands are assignable to LPy species. The occurrence of the two bands at two different frequency values (1623 and 1595 cm^-1^) may indicate that Lewis acid sites involved assume two different acidity strengths (Zaki et al. 1989).

Spectra taken from Py adsorption on 5CeAl, 10CeAl and 20CeAl samples at 300 K, show common feature of a gradual weakness in the intensity of the bands characteristic of the pure alumina. Py adsorption over phosphated alumina modified with ceria with different loading levels show similar spectra with phosphate free samples, so we only mention only one (xCeAl samples) to prevent the recurrence.

### Isopropanol decomposition

Preliminary experiments showed that the decomposition of isopropanol on the studied oxide catalysts proceeds through simultaneous dehydration and dehydrogenation reactions (at ≥ 470 K). Thus, the decomposition of isopropanol over the catalysts under study was carried out at 470 K. The obtained results are listed in Table 2 in terms of conversion%, acetone and propene selectivity%.

**Table 2:** **Conversion% of isopropanol as well as propene and acetone selectivities at 470 K**

Sample	Conversion%	Propene selectivity	Acetone selectivity
		%	%
Al	79.2	96.5	3.5
Ce	62.6	12.5	87.5
5CeAl	75	89.8	10.2
10CeAl	76.2	83.6	16.4
20CeAl	74.5	74.4	25.6
AlP	82.5	95.4	4.6
CeP	70.6	22.4	77.6
5CeAlP	86.5	94.8	5.2
10CeAlP	90.2	92.5	7.5
20CeAlP	88.7	90.1	9.9

The results obtained from decomposition of isopropanol on γ-Al_2_O_3_ (Table 2) display mainly conversion of isopropanol to propene, whereas the formation of acetone is found to be very low (3.5%). The obtained results indicated that the selectivity of propene is 96.5%, thus, alumina surface have strong acidic sites, which is evident from the higher dehydration process than dehydrogenation, these results agree with that in literature (Khalaf et al. 2007). Alumina surface is known to acquire strong Lewis acid sites, which explain the high activity towards the dehydration pathway (Waqif et al. 1992). The results for pure ceria show that the dehydrogenation (87.5%) of isopropanol to acetone is higher than the dehydration (12.5%) process indicating the basicity of ceria surface. Ceria modified alumina samples, xCeAl, show that the incorporation of ceria into alumina resulted in a decrease in the propene selectivity from 96.5% for alumina to 74.4% for 20CeAl. This descending in propene selectivity% is analog with ascending of acetone selectivity% and could be attributed to the basicity of ceria. (Zaki et al. 1997) reported that ceria surface has Lewis base sites assuming a strong nucleophilic reactivity.

At the same time, the conversion of isopropanol and the selectivity towards propene formation for the phosphate catalysts, xCeAlP, is higher than that for the phosphate free catalysts. Knowing that isopropanol dehydrate to propene over acidic catalysts, this could explain such behavior of the phosphated catalysts. Furthermore, the obvious increase in the catalytic activity (% conversion) of the phosphated catalysts than phosphate free samples is in accordance with the data obtained from surface area measurement, in which the S_BET_ for xCeAlP > S_BET_ for xCeAl.

## Conclusions

In conclusion, the addition of ceria and/or phosphate onto alumina with different loading levels causes a modification in the surface texture and porosity. The results show also that the ceria added to alumina is well dispersed on the surface of alumina and has no effect on the phase structure of pure alumina (γ-Al_2_O_3_ structure). Alumina, ceria and ceria-alumina composite show Lewis acid sites with different acidity strengths. Isopropanol decomposed to mainly propene by pure alumina and the addition of ceria decreases the amount of propene selectivity% and increases the acetone selectivity%, this is due to the basicity of ceria. Moreover, the phosphated samples have higher propene selectivity% than phosphate free samples indicating that the phosphate ions increase the acidity of the samples.

### Experimental

#### Materials

Two series of the catalysts under studying have been prepared. The first series is ceria doped alumina catalysts, abbreviated as xCeAl (where, x = 5, 10 and 20 wt%wt ceria) were prepared by coprecipitation method. The 1.5 molar solutions of Al(NO_3_)_3_.9H_2_O and Ce(NO_3_)_3_∙6H_2_O were prepared separately and then mixed in a volume proportions according to the final desired composition of catalysts. The resulting solution was stirred and heated to 350 K in a round bottom flask. The aqueous 1:1 M NH_4_OH solution was added drop wise to the nitrate solution under vigorous stirring until pH 8 was attained. After ageing for two hours, the excess solution was removed by filtration. Precipitate was washed repeatedly by distilled water followed by drying at 380 K for 12 h. The dried precursor was crushed to fine powder and calcined in the presence of static air at 873 K for 3 h.

The second series, xCeAlP (P refers to phosphate and x is the loading level of ceria, 5, 10 and 20 wt%), was obtained by impregnation of an aqueous solution of the impregnating (NH_4_)_2_HPO_4_ solution and held stirring for 1 h. The solution was adjusted to give 6 Wt% PO_4_^-3^ content. The excess water present was removed by evaporation at 380 K. The catalysts were obtained by calcination of the dried samples at 873 K for 3 h.

### Apparatus and techniques

#### Thermal analysis

Thermogravimetric analysis (TGA) and deferential thermal analysis (DTA) were performed between room temperature and 1273 K in a static atmosphere of air, using Linseis STA PT 1600 thermogravimetric analyzer. The rate of heating was standardized at 10 K /min., and small portions (5–15 mg) of the sample were used in TG measurements.

#### X-ray powder diffractometry

XRD diffractograms were recorded for all samples using a model JSX-60PA JEOL diffractometer (Japan) using Cu Kα radiation (λ = 1.5418 Å). The generator was operated at 35 kV and 20 mA. The samples were scanned in the range of 2θ = 10–70° at a scanning speed of 6° min^-1^. For identification purposes, diffraction patterns (I/Iº) versus *d* spacing (Å) were matched with the relevant ASTM standards (Frank et al. 1981). The crystallite size *D* of the samples were calculated using the Scherrer relationship (Klug & Alexander 1970):

1 $$D=\frac{\mathrm{K\lambda}}{\beta \cos\theta }$$

where *K* is the crystallite shape constant (≈1), *λ* the radiation wavelength, *β* the line breadth (radians) and *θ* is the Bragg angle.

#### Nitrogen adsorption isotherm measurement

Full nitrogen adsorption/desorption isotherms at 77 K were obtained using a NOVA 2200, version 6.10 high-speed gas sorption analyzer (Quantachrome Corporation USA). The calcined samples were first out-gassed at 470 K for 1 h. Twenty four-point adsorption and desorption isotherms were obtained, from which *BET* surface areas were derived using standard and well-established methods (Sing et al. 1985; Webb & Orr 1997).

#### FTIR spectra for pyrodine adsorption

For pyridine (*Py*) adsorption on test samples a wafer of 15–20 mg/cm^2^ were mounted in a Pyrex vacuum cell fitted with CaF_2_ windows. The samples were pretreated at 700 K for 1 h in stream of O_2_ followed by evacuation at 700 K for 1 h, then cooled to room temperature to obtain the background IR spectra using Thermo scientific Nicolet 380 FTIR spectrophotometer. Then, 5 Torr *Py* (1 Torr = 133.32 Pa) were admitted at 300 k for 5 min., degassed for 5 min. at this temperature in order to remove the fraction physisorbed *Py*, and the spectra were then taken at room temperature.

#### Catalytic activity (isopropanol decomposition)

The catalytic activity experiments for isopropanol decomposition were carried out in a fluidized bed quartz flow reactor at atmospheric pressure. 0.2 g of the catalyst was activated in-situ at 670 K for 1 h in N_2_. isopropanol (Merck, product, 99.9%) was introduced at a flow rate of 15 ml min^-1^ into carrier gas flow of N_2_. The reaction products were analyzed by gas chromatography on 2-m long 1/8״ column packed with 10% Carbowax and Chromm WHP 80/100 using a model 3400 Varian Gas Chromatograph equipped with a flame ionization detector (FID).

## Authors’ information

Assoc. Prof. Hussein A. Khalaf: He was born in 1970 in Minia, Egypt. He received a B.Sc. degree of pure chemistry from Faculty of Science, Minia University, Egypt, 1992. He obtained a M.Sc.(1999) and Ph.D (2005) degrees in Physical chemistry (surface and catalysis) from Minia University, Egypt, under the supervision of Professors Seham A. Mansour, Nasr E. Fouad, Ahmed K. Nohman and Gamal A. Mekhemer. And he is currently working as associated prof. at Omar El-Mukhtar University, Libya.

## Authors’ original submitted files for images

Below are the links to the authors’ original submitted files for images. Authors’ original file for figure 1 Authors’ original file for figure 2 Authors’ original file for figure 3 Authors’ original file for figure 4 Authors’ original file for figure 5 Authors’ original file for figure 6
