# Supplementary figures and images for: The negative effect of ceria on the propene selectivity for isopropanol decomposition over phosphated and phosphate-free ceria/alumina catalysts
Source: Springerplus. 2013 Nov 20;2(1):619. doi: 10.1186/2193-1801-2-619 (PMC3858591; doi:10.1186/2193-1801-2-619)

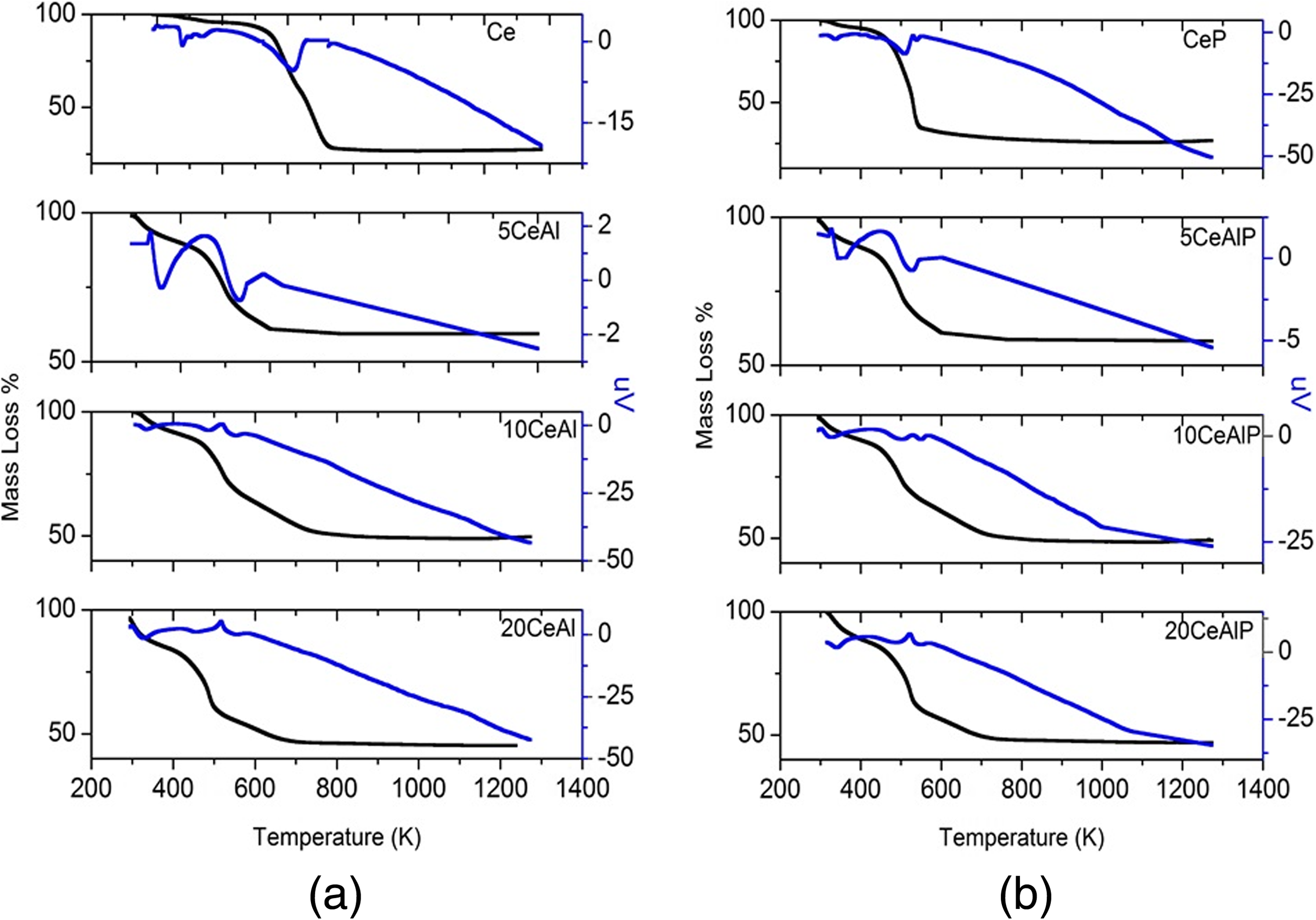

Supplement: Supplementary file 1 — Authors’ original file for figure 1 [file 40064_2013_684_MOESM1_ESM.tiff]

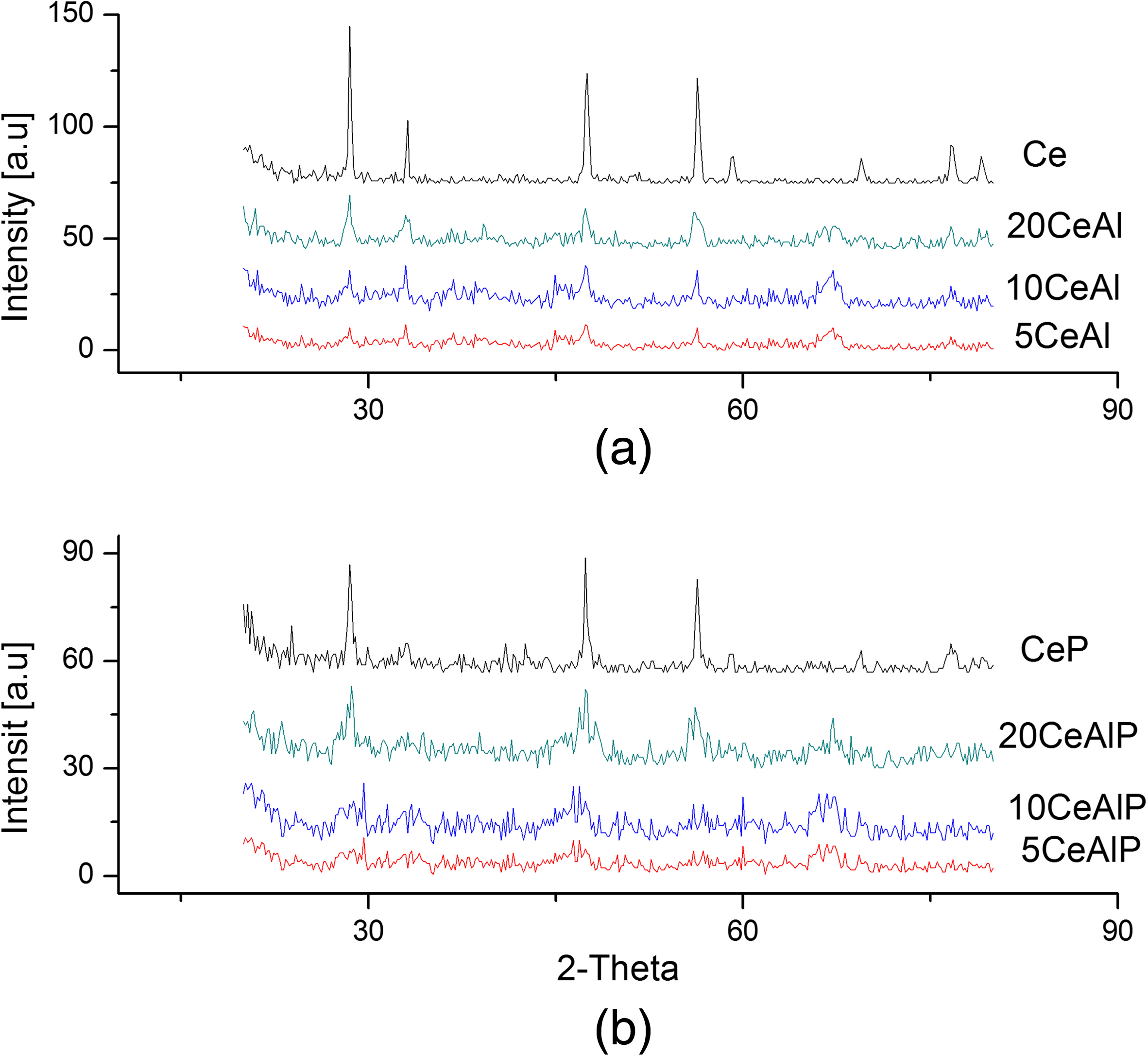

Supplement: Supplementary file 2 — Authors’ original file for figure 2 [file 40064_2013_684_MOESM2_ESM.tiff]

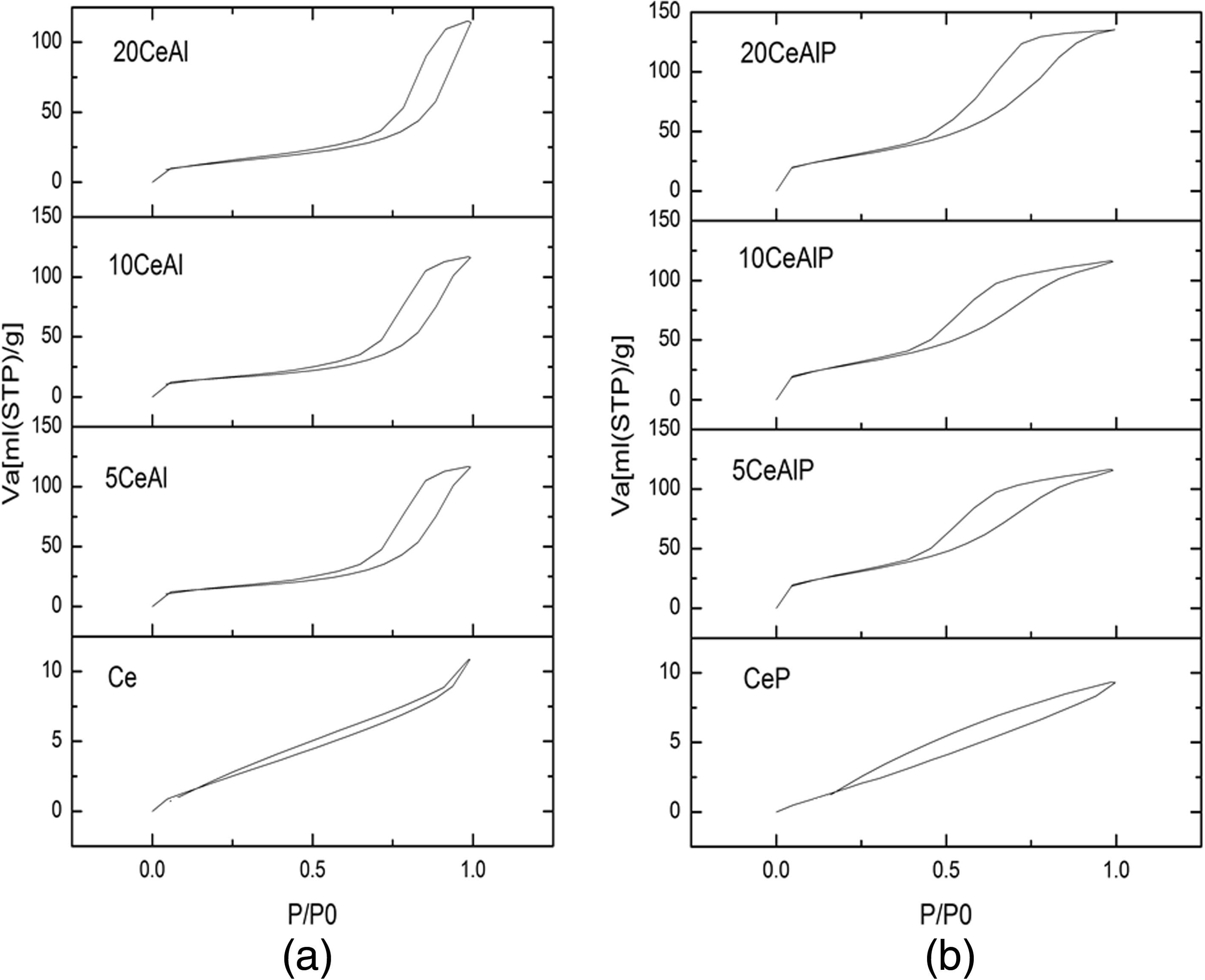

Supplement: Supplementary file 3 — Authors’ original file for figure 3 [file 40064_2013_684_MOESM3_ESM.tiff]

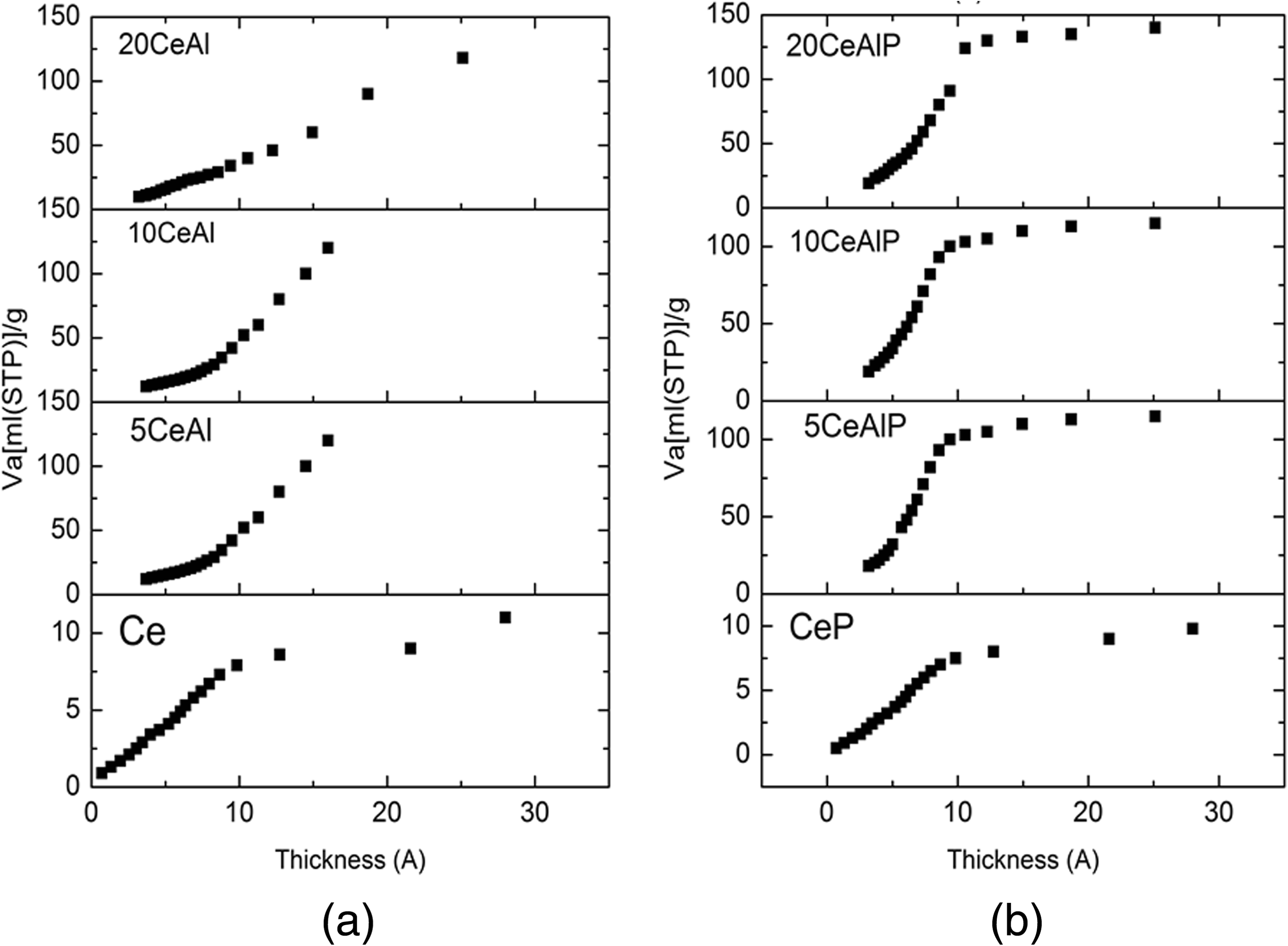

Supplement: Supplementary file 4 — Authors’ original file for figure 4 [file 40064_2013_684_MOESM4_ESM.tiff]

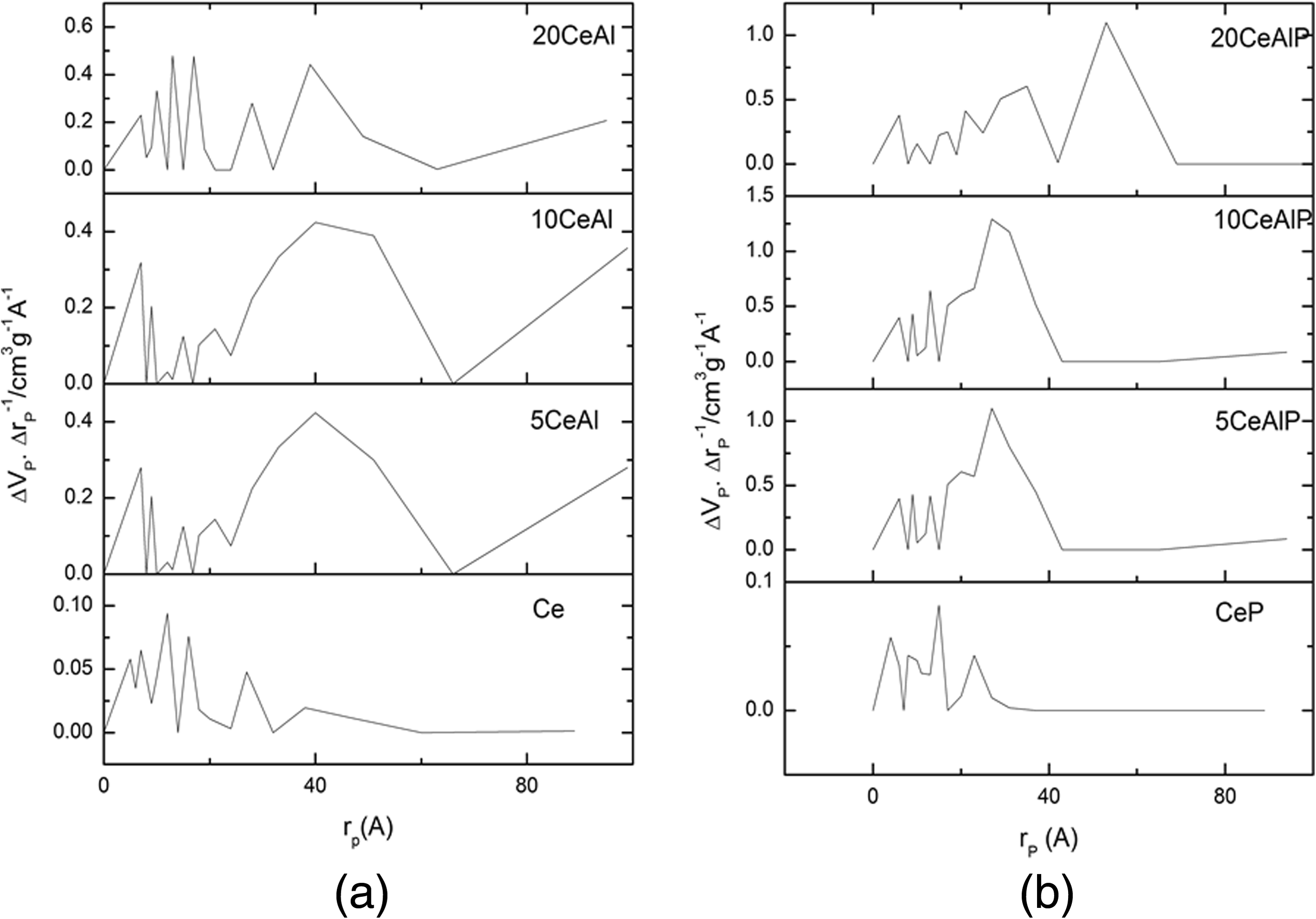

Supplement: Supplementary file 5 — Authors’ original file for figure 5 [file 40064_2013_684_MOESM5_ESM.tiff]

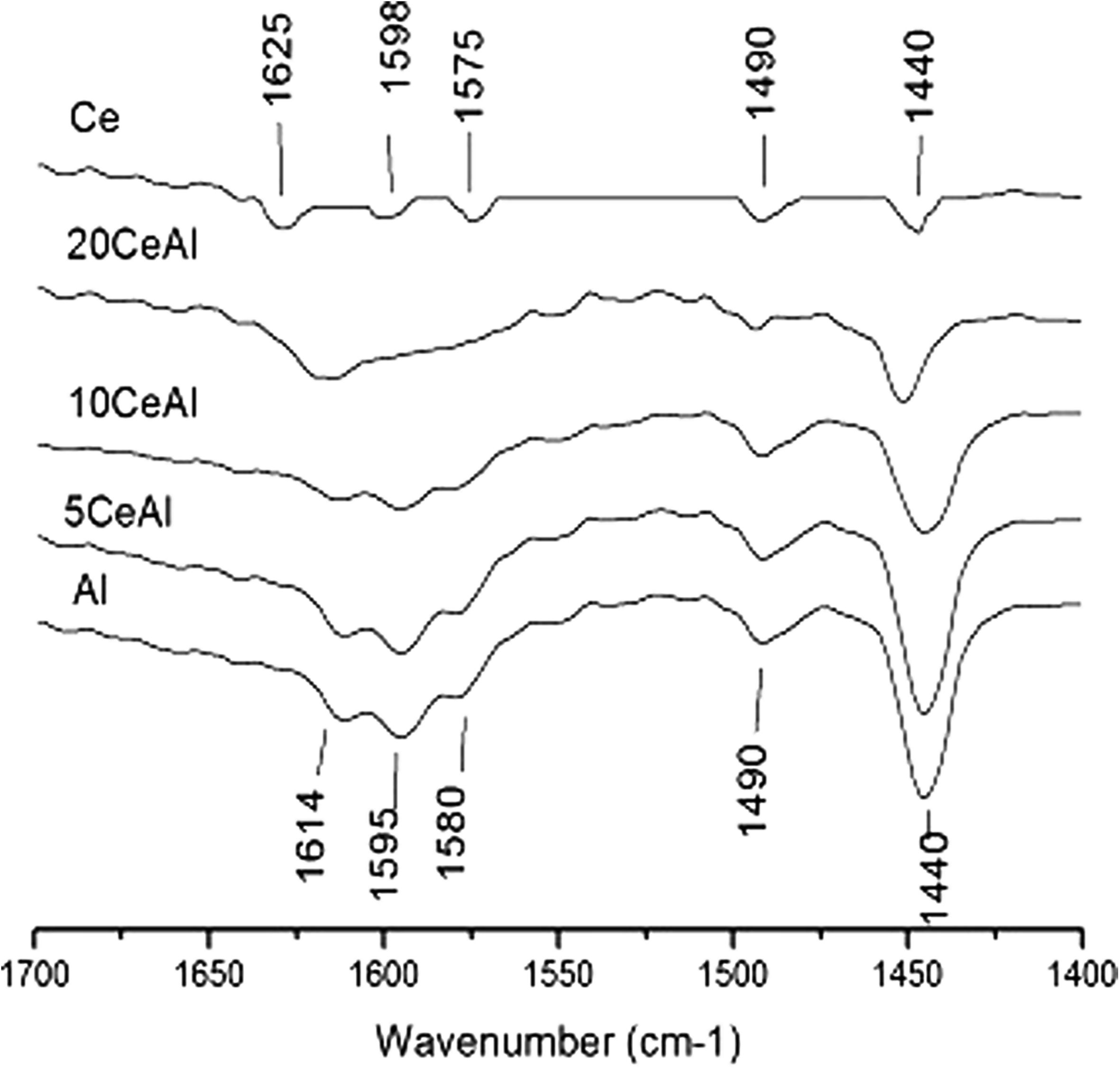

Supplement: Supplementary file 6 — Authors’ original file for figure 6 [file 40064_2013_684_MOESM6_ESM.tiff]
